# Editorial: Advanced nanotechnological detection and drug delivery configurations

**DOI:** 10.3389/fphar.2023.1198100

**Published:** 2023-04-13

**Authors:** Seyyed Alireza Hashemi, Mohammad Arjmand, Kamran Bagheri Lankarani, Wei-Hung Chiang, Seeram Ramakrishna

**Affiliations:** ^1^ Nanomaterials and Polymer Nanocomposites Laboratory, School of Engineering, University of British Columbia, Kelowna, BC, Canada; ^2^ Health Policy Research Center, Health Institute, Shiraz University of Medica Sciences, Shiraz, Iran; ^3^ Department of Chemical Engineering, National Taiwan University of Science and Technology, Taipei, Taiwan; ^4^ Department of Mechanical Engineering, Center for Nanofibers and Nanotechnology, National University of Singapore, Singapore, Singapore

**Keywords:** detection, drug delivery systems, nanomedicine, treatment, diagnostic

The widespread of infectious diseases and the advent of complex illnesses because of the human lifestyle led to considerable human pathogenesis and subsequent mortality, leaving a massive loss on the shoulder of human society (Gholami et al., 2020; Hashemi et al., 2021c). Even with the considerable investment of healthcare systems in sensitive detection approaches and wise treatment strategies, the world still encounters the flaw of fast person-to-person transferable pathogens that sometimes surpass cardiovascular diseases and cancer. This matter necessitates systematic investigations in the discovery of novel drugs with low side effects and sensitive detection approaches in a highly specific manner (Mousavi et al., 2019; Hashemi et al., 2021a; Hashemi et al., 2021b; Hashemi et al., 2022a; Hashemi et al., 2022b).

This Research Topic entitled “*Advanced Nanotechnological Detection and Drug Delivery Configurations*” is established with the aid of five expert editors with expertise in different disciplines toward promoting the current knowledge of drug discovery and advanced detection approaches. The Research Topic was prosperous, and five articles, including four original research articles and one review paper, were published after a rigorous peer review process supported by expert reviewers in the field.

Summarizing the published original research articles in the Research Topic, Dhondt et al. unveiled the contribution of fluid therapy in developing augmented renal clearance (ARC) in piglet models. Their study focused on the fluid administration of renal function by employing a piglet animal model to quantify the effect of fluid administration on the pharmacokinetic effects of the renally excreted drugs. Their study suggested the key impact of fluid therapy on the key factors of the ARC that should be considered upon the administration of the renally excreted drugs.

Huang et al. summarized and evaluated 10 years of progression in the field of gene therapy, focusing on the usage of nanomedicine and the problems in their transition into active use. Their study suggested the potential impact of several technologies in the case of targeted drug delivery. Accordingly, the systematic evolution of ligands by exponential enrichment (SELEX) and aptamer systems were found to be potent drug delivery configurations. The smart gene editing through clustered regularly interspaced short palindromic repeats (CRISPR)-associated protein 9 (Cas9) is recognized as another method of target-oriented and specific disease treatment that showcased promising results in cancer therapy. The exosomes and their derivatives were also suggested as promising intelligent drug delivery systems for treating complex illnesses. This comprehensive study is a useful tool for decision-makers to put effort into the most practical strategies for specific and efficient disease treatment.

Zhu et al. correlated the ion channel gene signatures with the prognosis, diagnosis, and individualized treatments in patients with clear cell renal cell carcinoma (ccRCC). For this case, they extracted the RNA profiles, clinical data of patients with ccRCC, and public databases to propose a proper diagnosis approach and timely treatment techniques upon monitoring the level of ion-channel-related genes, known as specific biomarkers toward monitoring the typical structure of the kidney. Xian and co-workers (Xiang et al., 2022) investigated the potential of Ligusticum Chuanxiong as a traditional Chinese medicine against Osteoarthritis using network pharmacology and molecular docking. These two investigations highlighted the potential of in-depth data analysis in designing and finding the most effective treatment technique and/or diagnosis approach for complex illnesses with fewer clinical and animal trials.

In a review study published in the Research Topic, Ge et al. reviewed the potential of the PEGylated lipid-based configurations for small interfering RNA (siRNA) as a potential treatment tool. Based on their review, the long-circulating lifetime, biocompatibility, low toxicity, and ease of scale-up made the PEGylated lipid-based configurations versatile delivery systems for nucleic acids. They also investigated the recent progress in the case of these exciting drug delivery systems and provided the challenges they encounter and possible strategies to address them.
